# A Clinical Study on Initial Experience of COVID-19 ARDS in Obstetric Patients at a Tertiary Care Centre in India

**DOI:** 10.1155/2021/5591041

**Published:** 2021-03-29

**Authors:** Sheeba Marwah, Reenu Kanwar, Shahida Naghma, Anjali Dabral, Nitesh Gupta

**Affiliations:** ^1^Department of Obstetrics and Gynaecology, New Delhi 110029, India; ^2^VMMC and Safdarjung Hospital, New Delhi 110029, India; ^3^Department of Pulmonary, Critical and Sleep Medicine, New Delhi 110029, India

## Abstract

Coronavirus disease 2019 (COVID-19) is caused by a novel coronavirus (severe acute respiratory syndrome coronavirus 2 (SARS-CoV-2)) which causes severe viral pneumonia rapidly leading to acute respiratory distress syndrome (ARDS). Pregnant women are considered more vulnerable to severe viral respiratory infections owing to the physiological changes in pregnancy. In COVID-19, patient can present with a variety of symptoms of which dyspnoea is one that is also commonly seen in the late stages of pregnancy. The clinical presentation as well as response to therapy is highly variable, and since no conclusive proven treatment is available yet, prevention and symptomatic treatment remains the mainstay of management. Thus, we report a case series of four SARS-CoV-2-positive obstetric patients who presented with severe ARDS in a tertiary care hospital, posing diagnostic and therapeutic challenges to the clinician, and were managed with a holistic multidisciplinary stepwise approach. Through this, an effort has been made to sensitize the attending obstetrician on diverse presentation of COVID-19 disease and to emphasize the importance of prevention, early pick up, and timely optimal management of pneumonia in pregnant females with COVID-19. The clinical presentation of respiratory illness due to SARS-CoV-2 in pregnancy can be mistaken for exaggerated physiological changes of pregnancy leading to delay in seeking medical care. During the current pandemic, high suspicion for COVID-19 should be kept. If found symptomatic, immediate care should be sought in a designated facility and managed accordingly preferably with a multidisciplinary approach.

## 1. Introduction

The present COVID-19 pandemic with a worldwide spread is caused by SARS-CoV-2. Coronavirus disease is believed to be a zoonotic disease, with the intermediate host being unclear [1]. It is commonly transmitted through air, respiratory droplets, contact, and even fecal-oral route [2]. Coronavirus disease can present with fever, cough, and dyspnoea often followed by pneumonia. The World Health Organization (WHO) has termed the pneumonia caused by SARS-CoV-2 as the coronavirus disease 2019 (COVID-19) [3, 4].

About 80% of COVID-positive patients are classified as mild and the remaining 20% as severe or critical [5, 6] which are associated with complications like ARDS (acute respiratory distress syndrome), disseminated intravascular coagulopathy, renal failure, secondary bacterial pneumonia sepsis, shock with organ failure, and respiratory failure. Pregnant women are considered more vulnerable to severe viral respiratory infections. Till date, no treatment has been proven to be curative for the SARS-CoV-2 virus; hence, supportive treatment with supplemental oxygen therapy (if required) is the key for management.

The review of literature till date illustrates no additional risk in the morbidity and mortality associated with COVID-19 in pregnant women, but owing to the physiological changes in pregnancy like diaphragm elevation, increased oxygen consumption, and oedema of respiratory tract mucosa; they become intolerant to hypoxia [7–10]. In addition, hypercoagulable state in pregnancy increases the risk of pulmonary microvascular thrombosis. Due to all these factors, any superimposed insult by the virus may contribute to exacerbations of respiratory diseases in the antenatal and postpartum period. Because of similarity in exaggerated physiological symptoms in normal pregnancy and in any respiratory illness, there might be a possible delay in seeking medical care by patients during pregnancy. This topped with variable presentation of COVID-19 pneumonia, which can easily be misleading to the attending clinician. Thus, an attempt is made to describe a case series of four obstetric patients admitted in a tertiary hospital, emphasizing the importance of prevention, early diagnosis, and management of COVID pneumonia in these cases.

## 2. Case Presentation

### 2.1. Case 1

**Figure 1:**
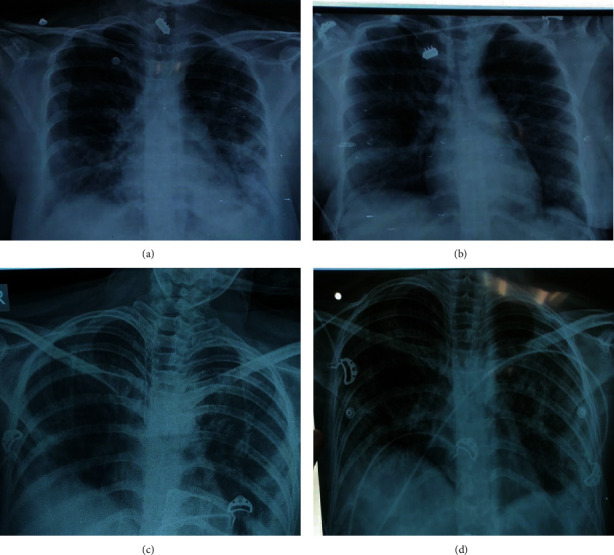
Radiological findings: (a) postnatal day 7, (b) postnatal day 11, (c) postnatal day 30, and (d) postnatal day 36.

A 26-year-old female P1L1 (para 1, living 1) with no previous comorbidities had full-term normal vaginal delivery. Her antenatal period was thoroughly supervised and uneventful. She was completely asymptomatic till day 5 of postpartum period, when she presented with shortness of breath with dry cough for 2 days. She was referred to our tertiary care hospital for further management in view of clinically deteriorating symptoms. At the time of presentation in emergency room (ER), she had fair general condition: pulse—120/min, BP (blood pressure)—104/70 mmHg, RR (respiratory rate)—36/min, SPO_2_ (oxygen saturation)—64% on room air, cardiovascular system—normal, chest ex—B/L (bilateral) crepitations present, B/L decreased breath sounds, P/A (per-abdomen)—uterus 16 weeks, well contracted, nontender, P/V (per vaginum)—episiotomy healthy, lochia healthy. On reviewing her records, chest X-ray (done 24 hrs back) showed patchy peripheral extensive consolidation along with ground-glass opacities in B/L lung fields predominantly in mid and lower zones suggestive of atypical pneumonia. ECG (echocardiograph) showed sinus tachycardia. After initial management and evaluation, she was admitted in SARI (severe acute respiratory distress isolation) ward in collaboration with Medicine Department. All routine investigations, cultures, and ABG (arterial blood gas analysis) were sent. COVID-19 testing was done using nasopharyngeal swab. She was tested positive for SARS-CoV-2 and was shifted to designated COVID-19 ICU (intensive care unit). Patient was put on NRBM (nonrebreathing mask) at 15 lt/min followed by NIV (noninvasive ventilation). Because of inability to maintain oxygen saturation, patient required intubation and was put on mechanical ventilation for a day. She was provided with supportive care guided by Government of India (GOI) guidelines, including the antiviral drug (remdesivir) and higher intravenous antibiotics with steroid therapy for 10 days. She was extubated on the very next day and was put on NIV again. Her blood reports were within normal range except indicative of infection: haemoglobin—12.2 gm/dl, TLC (total leucocyte count)—16,300, platelets—1,13,000, INR (international normalized ratio)—1.18, LFT/KFT (liver function tests/kidney function tests)—normal. Ultrasound chest was done on day 10 suggestive of B/L consolidation in left to right and no evidence of any effusion. During her hospital stay, she complained of pain left-sided abdomen. She was evaluated in conjunction with gastroenterologist. An ultrasound of the whole abdomen was done and she was diagnosed to have portal cavernoma, which did not require any active intervention. In view of persistent tachycardia, cardiology consultation was done. Her 2D echo (2-dimensional echocardiogram) revealed ejection fraction—55%, no clots, no RWMA (regional wall motion abnormalities). She was subjected to serial chest radiographs, as advised by pulmonologist, which showed a gradual resolution in sizes of patches and opacity as shown in Figures 1(a)–1(d).

There was no history of any contact with the COVID-19-positive patient; her newborn baby was also tested positive for SARS-CoV-2 and required admission in nursery but was discharged after 7 days. Her remaining contact tracing was negative. After almost 7 weeks of hospital stay, she was tested negative for COVID-19 disease in 2 consecutive reports. Due to the massive spread of infection to lower respiratory tract, the patient was required with NRBM for maintaining saturation for almost 2 months but severity of symptoms decreased significantly. She gradually started maintaining oxygen saturation on room air. At the time of writing this paper, the patient has been discharged back home in good health to join her family.

### 2.2. Case 2

A 34-year-old woman G2P1L1 (gravida 2, para 1, living 1) at 39 weeks of POG with previous LSCS was presented in the Gynaecology Casualty with complaints of fever, cough, and breathlessness for 5 days. She had an uneventful antenatal period with no previous respiratory/cardiac disease or any history of high BP records. At the time of presentation, her general condition was dyspnoeic: pulse—130/min, BP—160/112 mmHg, urine albumin—+3, temperature—98.3°F, RR—24/min, SPO_2_—94% (room air), CVS—normal, chest examination—B/L crepitations present, B/L decreased breath sounds, on P/A examination—36 wks, cephalic, foetal heart sound (FHS) absent, uterus relaxed, no scar tenderness, nontender, P/V—1 cm dilated, uneffaced, membrane present, vertex—3, pelvis adequate.

In view of high BP with urine albumin +3 and crepitations in B/L chest, diagnosis of severe preeclampsia with pulmonary edema with differential of COVID-19 was made. She was put on oxygen mask with reservoir, injectable diuretics were given, and urgent ultrasound was done in ER which revealed intrauterine death. After initial stabilization in causality, patient was admitted in SARI Ward. She was put on NIV as she was not maintaining saturation (<90%) (haemoglobin—11.3 gm/dl, TLC—9300, platelets—1.75 lakhs/mm^3^, INR—1.02, KFT/serum electrolytes—normal, LFT—1/243/256/542). Chest X-ray was done which showed inhomogeneous airspace in peripheral distribution with B/L blunting of costophrenic angles likely due to pleural effusion. COVID-19 testing was done with nasopharyngeal swab on the day of admission which came negative. Patient went in spontaneous labor and was shifted to the labor room for monitoring of progress of labor where she required with NRBM to maintain oxygen saturation. She was started on antibiotics and antiviral drugs. Because of the persistent high blood pressure, cardiology opinion was sought. 2D echo was done which was essentially normal. As there was strong clinical suspicion of COVID-19 pneumonia, repeat testing was done after 2 days and patient was diagnosed to be SARS-CoV-2 positive. Patient was immediately shifted to a COVID-19-designated ICU. She went in spontaneous labor and delivered a fresh still born baby girl of 2.45 kg. There was no intrapartum complications. Postnatally, further serial chest X-rays showed radiological improvement in the form of decrease in pleural effusion and clearing of costophrenic angles. After delivery, she required high flow oxygen for almost a week following which she was gradually shifted to oxygen mask and started maintaining oxygen on a room air. After being asymptomatic for a week, patient was discharged for further home isolation as per GOI guidelines with an advice to follow up after 2 weeks or SOS.

### 2.3. Case 3

A 30-year-old female G2P1L1 at 37 weeks and 2 days of gestation with gestational hypertension was presented in the Gynaecology Casualty of our hospital with complaints of breathlessness for 2 days. Patient was referred from a government hospital in view of breathlessness with pulmonary edema. Her antenatal period was uneventful. At the time of presentation, patient was visibly dyspnoeic: pulse—118/min, BP—170/122 mmHg, urine albumin—+3, temperature—afebrile, RR—28/min, SPO_2_—96% (room air), CVS—normal, chest examination—B/L crepitations present, B/L decreased breath sounds, on P/A examination—34 weeks, cephalic, FHS present and regular, uterus relaxed, P/V—Os closed, uneffaced, pelvis adequate. Diagnosis of severe preeclampsia with pulmonary edema was made, and patient was initiated on diuretic while being on high flow oxygen. She was prepared for emergency LSCS (lower segment caesarean section) under a high-risk consent. COVID-19 testing was done preoperatively. Emergency LSCS was done under general anaesthesia. A baby boy of 1.9 kg was delivered and was shifted to NICU (neonatal intensive care unit) in view of low respiratory efforts and low birth weight. Baby was tested negative for COVID-19 disease. An attempt to extubate the patient was made postsurgery but could not be done in view of poor chest condition. She was shifted to ICU in immediate postoperative period. Injectable antibiotics was given along with diuretics. Her COVID-19 test came back positive. She was shifted to COVID-19 ICU where the patient was given supportive care in the form of mechanical ventilation, antibiotics, and obstetrics postop care. Her baby was also tested positive and was managed in NICU. She was extubated on the next day and was put on NIV. After 3 days, she was able to maintain oxygen saturation without support. She was later shifted to the Gynaecology COVID-19 ward and discharged from there after 10 days with a baby boy.

### 2.4. Case 4

A 26-year-old female G2P1L0 (gravida 2, para 1, living 0) at 37 weeks and 5 days of POG with hypothyroidism was presented in the Gynaecology Casualty with complaints of leaking per vaginum for 12 hrs. There were no complaints of fever, cough, and breathlessness. At the time of presentation, her general condition was fair: pulse—86/min, BP—112/76 mmHg, temp—97.6°F, RR—24/min, SPO_2_—94% (room air), CVS—S1S2+, chest ex—B/L clear, on P/A ex—34 wks, cephalic, FHS present, and regular (140/min), uterus contraction present, P/V—2 cm dilated, 50% effaced, membrane absent, vertex—2, pelvis adequate.

She was admitted in the labor room where she progressed gradually and spontaneously and had full-term normal vaginal delivery of a baby boy of 2.4 kg. The baby and mother were doing absolutely fine until postnatal day 2, when she started having difficulty in breathing and a cough (on examination, pulse—100/min, BP—132/90 mmHg, SPO_2_—94%, temp—100.8°F, heart—normal, no murmur, chest ex—B/L crepitations present). She was managed immediately with oxygen support, diuretics, and analgesics. A vital monitor was attached. Her COVID-19 testing was done, and meanwhile, the patient was kept in isolation. Cardiology referral was done. ECG was found to be normal, and pro-BNP (brain natriuretic peptide) came out negative. Her lab reports were as follows: Hb—13.5 gm/dl, TLC—11,500, PT—1,70,000. Her chest X-ray showed B/L consolidation predominantly in lower zones with no evidence of pleural effusion. She came out COVID-19 positive and was shifted to a designated COVID-19 ICU as she was not maintaining oxygen saturation with an oxygen mask at 10 lt/min. In ICU, she was put on NRBM for 2 days along with multivitamins and zinc supplements. Taking antibiotics was continued for 5 days. After 5 days of diagnosis, she started maintaining oxygen saturation on room air with radiological improvement in serial X-rays. Patient was moved back to the COVID-19-designated ward. Her baby tested negative for COVID-19. At the time of writing this paper, patient is still admitted in the hospital with no symptoms. She is planned to be discharged after testing negative.

## 3. Discussion

The ongoing COVID-19 pandemic is a global public health emergency, the course and outcome of the disease being highly unpredictable. Illness caused by SARS-CoV-2 ranges from asymptomatic viraemia to symptomatic disease similar to influenza like illness in various stages and can even be fatal. Also, the morbidity associated with physiology of pregnancy overlapping with respiratory illness leads to deferment in seeking timely help. Therefore, early diagnosis and critical management of these women becomes an important confounding factor for associated comorbidity in such patients. Over the past few months, lots of insights have been gained on COVID-19, but robust evidence on respiratory symptoms of pregnant women with SARS-COV-2 are unavailable; hence, we present a narrative case series of 4 young females affected with SARS-CoV-2 during pregnancy. A comparative evaluation of present series with the limited body of literature available in current times has been discussed (Table 1).

Recent studies have revealed negligible difference in presentation of COVID-19 infection in pregnant and nonpregnant women. However, associated comorbidities especially hypertensive disorders of pregnancy can worsen the stages of COVID-19 and thus their outcome. This is consistent with the study by Chen and colleagues, wherein the clinical characteristics reported in pregnant women with confirmed COVID-19 infection were similar to those reported for nonpregnant adults with confirmed COVID-19 infection in the general population and were indicative of a relatively optimistic clinical course and outcomes for COVID-19 infection [7, 10].

However, contact with a positive patient is a known risk factor for acquiring SARS-CoV-2 virus but it was absent in all the cases in the current study. Similarly, the observations reported by Sahin et al. wherein they found that 75% of the patients had negative history of close contact. This reiterates the importance of high clinical suspicion of COVID-19 in the presence of clinical findings even without significant history of exposure [11].

The presenting symptoms in the patients under current study included fever, cough, cold, chest pain, shortness of breath, and myalgia which were consistent to the findings of previous researchers [11, 12].

Even though in-house HRCT for SARS-CoV-2-positive patients was not available, all four women in the current series underwent chest X-rays which revealed peripheral patchy infiltrates in B/L lungs. This finding was peculiarly parallel to the most frequent CT finding reported by most of the investigators in COVID-19 pneumonia in pregnancy [5, 12]. This highlights the importance of adopting a step-by-step approach to the management of dyspnoea in pregnancy including all preliminary investigations like chest radiographs even in modern times.

Inflammatory markers (serum ferritin, interleukin 6, C-reactive protein, and serum LDH) were increased in all 4 women. This is in synchrony with several other studies and adding to the evidence in the role of anti-inflammatory agents in COVID-19 disease, thus emphasizing the importance of proinflammatory cytokines in disease progression [13].

Amongst the four patients reported in our series, two presented with findings consistent with COVID-19 pneumonia. While the other two of them presented with acute respiratory distress syndrome owing to pulmonary edema. The largest case narrative on COVID-19 pneumonia in pregnancy, conducted in Spain, showed that pregnant females have a 61.5% chance of developing pneumonia as compared to the general population between age groups 30-40 years [14]. Since the advent of novel SARS-CoV-2, only few isolated cases of COVID-19 pneumonia in pregnancy have been reported. It is associated with varied complications including acute respiratory distress syndrome, disseminated intravascular coagulopathy, renal failure, secondary bacterial pneumonia sepsis, shock with organ failure, respiratory failure requiring mechanical ventilation, or refractory hypoxemia requiring extracorporal membrane oxygenation. However, such serious complications could be averted successfully in our patients due to vigilant monitoring and timely intervention due to high index of suspicion.

All of the four patients in the series were successfully discharged; this was in congruence with a systematic view of 41 pregnancies affected by COVID-19 and another report of 108 pregnancies, which also reported no maternal deaths [15, 16]. However, a different observation was made by an Iranian case series wherein they reported 7 maternal deaths in nine pregnant women with critical COVID-19 [17].

Out of 4 cases included in the current study, 3 had live births; whereas, one was a diagnosed intrauterine death. Out of these, only one baby (Case 1) was tested positive who was also discharged after 7 days in a healthy condition. However, this could be a horizontal spread and so vertical transmission could not be documented in this particular case due to the absence of breast milk, histopathological examination of placenta, cord blood, and amniotic fluid. The largest systemic review and meta-analysis studying the vertical transmission in coronavirus positive cases included 39 studies (1316 pregnant women) and also concluded no transmission of COVID-19 from the mother to the foetus in utero till date [18].

There is no evidence-based treatment for COVID-19-associated pneumonia yet, and the treatment mainly focuses on supportive therapy, improving immunity and supplemental oxygen therapy. All four patients included in the study were given steroid since day 1 of admission as per protocol and government of India guidelines and they responded well to the therapy. This is alike the inference of RECOVERY trial which depicted that steroids significantly decrease the 28-day mortality for individuals with COVID-19-positive patients requiring oxygen (age-adjusted rate ratio 0.83; 95% CI 0.75–0.93; *P* < 0.001) [19].

There is limited data available on the use and safety of remdesivir (an antiviral nucleotide prodrug that effectively inhibits replication of SARS-CoV-2 in vitro) in pregnancy. Although recent case reports documented no side effects of remdesivir in pregnant women, it cannot be concluded to be safe in pregnancy since all these women had administered the drug in the 2^nd^/3^rd^ trimester only [20–22]. Therefore, none of the women were given remdesivir as part of therapy in the current study in antenatal period. In another case series by Burwick et al., of 86 women (67 were pregnant, 19 have immediate postpartum), remdesivir was administered on a compassionate basis for severe COVID-19 infection and resulted in high clinical recovery, though with increased rate of preterm delivery [23].

Amoxicillin and azithromycin are classified as class B by FDA and commonly used in pregnancy and breastfeeding [24, 25]. All of the cases in current study with ARDS were given azithromycin as per institutional protocol. Although antibacterial therapy should be started, only if a bacterial infection is suspected, but the initiation was justifiable in our patients as prophylaxis against community-acquired pneumonia in all women with COVID-19 disease.

Symptomatic pregnant women should seek timely medical consultation and help, and women with clinical suspicion of infection should be isolated and investigated. Women diagnosed with infection should be promptly admitted to a negative pressure isolation ward, preferably in a designated hospital with adequate facilities and multidisciplinary expertise to manage critically ill obstetric patients. Adequate rest, hydration, nutritional support, and water and electrolyte balance should be ensured. It is essential to monitor vital signs and oxygen saturation closely. Depending on the severity of the disease, supplemental oxygen inhalation (60%-100% concentration at a rate of 40 l/min) should be given via high flow nasal cannula depending on the severity of hypoxemia. Intubation and mechanical ventilation or even extracorporal membrane oxygenation (ECMO) may be required to maintain oxygenation as was seen in two of our cases (cases 1 and 3).

## 4. Conclusion

COVID-19 is a global health threat because of its varied presentation and unpredictable behaviour. The outcome is multifactorial; hence, delay in seeking medical care can have adverse implications. Active infection in pregnancy may present with severe respiratory illness in antenatal and postnatal females owing to the physiological changes in the immune and respiratory system. Dyspnoea in pregnancy, often missed, is a grave complication of COVID-19 infection in pregnancy, which if picked up in time can result in optimal maternal and foetal outcome by avoiding serious complications. Diverse presentations and the need to suspect COVID-19 infection earlier in such patients should be emphasised to gynaecologists. Pregnant women being high risk should refrain from unnecessary travel, avoid crowded places, and more importantly, practice and maintain good personal and social hygiene. They should check their temperature regularly and immediately inform their doctor if they experience shortness of breath, cough, or fever. If found symptomatic, immediate care should be sought in designated facility with multidisciplinary approach. To date, research studies conducted in pregnant women with COVID-19 have showed only few maternal and neonatal complications [26], but more concrete evidence and further studies are required to gain insight on its behaviour in pregnancy as these studies included a small number of women over a short period of time.

## Figures and Tables

**Table 1:** 

S no	Studies	Study population	Type of study	Study centre	Observations
1.	Chen et al.	118 pregnant women	Epidemiological review	China	(i) 8%—severe disease (ii) 0.008%—required NIV (iii) Mortality—nil

2.	San-Juan et al.	52 pregnant women with COVID-19	Single-centred cohort study	Spain	(i) Pneumonia—61.5% (>50% required supplemental oxygen) (ii) Most common radiological findings were bilateral ground-glass opacities (46.8%) followed by bilateral or unilateral alveolar infiltrates (40.6%) (iii) ARDS—25% (iv) IMV—6.2%

3.	Knight et al.	427 pregnant women with COVID-19	Prospective population-based cohort study	All obstetric unit in UK	(i) Needed critical care—10% (ii) ECMO—1% (iii) Pneumonia on imaging—24%

4.	Kayem et al.	617 pregnant women with COVID-19	Case series	France	(i) Typical chest CT features of pneumonia—8.3% (ii) Nasal oxygen therapy—13.5% (iii) NIV—1.6% (iv) Invasive mechanical ventilation—4.7% (v) ECMO—1% (vi) Association with hypertensive disorders—3.4%

5.	Present case series	4 pregnant women with COVID-19-related ARDS	Case series	India	(i) Typical chest radiograph features of pneumonia—25% (ii) NIV—25% (iii) NRBM—25% (iv) Invasive ventilation—50% (v) Association with hypertensive disorders—50%
